# First-Generation Antihistamines and Seizures in Young Children

**DOI:** 10.1001/jamanetworkopen.2024.29654

**Published:** 2024-08-28

**Authors:** Ju Hee Kim, Eun Kyo Ha, Boeun Han, Taehwan Han, Jeewon Shin, Kyu Young Chae, Seonkyeong Rhie, Man Yong Han

**Affiliations:** 1 Department of Pediatrics, Kyung Hee University Medical Center, Kyung Hee University School of Medicine, Seoul, Korea; 2 Department of Pediatrics, Hallym University Kangnam Sacred Heart Hospital, Seoul, Korea; 3 Department of Pediatrics, CHA Bundang Medical Center, CHA University School of Medicine, Seongnam, Korea; 4 Department of Pediatrics, Yongin Severance Hospital, Yonsei University Health System, Yongin, Korea; 5 Department of Pediatrics, CHA Ilsan Medical Center, CHA University School of Medicine, Goyang, Korea

## Abstract

**Importance:**

The widespread use of antihistamines in children for treatment of common cold symptoms and their central nervous system effects, like drowsiness, underscore the importance of being aware of the associated risks.

**Objective:**

To assess associations between prescriptions of first-generation antihistamines and seizures in children using a comprehensive and nationwide dataset.

**Design, Setting, and Participants:**

This cohort study used a self-controlled case-crossover design. Data were obtained from the National Health Insurance Service database in Korea. Children born between January 1, 2002, and December 31, 2005, who visited the emergency department for seizure events (*International Statistical Classification of Diseases and Related Health Problems*,* Tenth Revision*, codes R56.8, G40, and G41) during the follow-up period were included. Follow-up was completed on December 31, 2019, and data were analyzed from June 3, 2023, to January 30, 2024.

**Exposure:**

First-generation antihistamine prescription.

**Main Outcomes and Measures:**

Primary outcome consisted of an index seizure event. Odds ratios (ORs) for seizure events were estimated using a conditional logistic regression model, comparing first-generation antihistamine prescription 1 to 15 days before seizure (hazard period) against control period 1 (31-45 days before the event) and control period 2 (61-75 days before the event) using the same period windows. Stratified analyses were conducted to examine the association with individual participant characteristics.

**Results:**

Of 11 729 children who had a seizure event, 3178 (1776 [55.9%] boys) were identified as having been prescribed antihistamines during the hazard or the control period, but not both. Seizure events were predominantly observed in children aged 6 to 24 months (985 [31.0%]) and 25 months to 6 years (1445 [45.5%]). During the hazard period, 1476 first-generation antihistamine prescriptions were recorded, in contrast to 1239 and 1278 prescriptions during control periods 1 and 2, respectively. After multiple confounder adjustments, first-generation antihistamine prescription was associated with an increased seizure event risk during the hazard period (adjusted OR [AOR], 1.22 [95% CI, 1.13-1.31]). Stratified subgroup analyses showed consistent results, particularly in children aged 6 to 24 months who were prescribed first-generation antihistamines having a higher risk (AOR, 1.49 [95% CI, 1.31-1.70]) than children aged 25 months to 6 years (AOR, 1.11 [95% CI, 1.00-1.24]; *P* = .04 for interaction). Furthermore, sensitivity analyses, including adjustment for exposure window periods, evaluation of new first-generation antihistamine prescriptions, comparison of control points from the same period 1 year prior, and exclusion of individuals using combination drugs, confirmed a similarly high risk.

**Conclusions and Relevance:**

In this cohort study, prescriptions for first-generation antihistamines were associated with a 22.0% higher seizure risk in children, especially in those aged 6 to 24 months. These findings emphasize the need for careful and judicious prescription of first-generation antihistamines in young children and underline the need for further research to elucidate associations between antihistamine prescriptions and seizure risk.

## Key Points

**Question:**

What is the association of prescriptions of first-generation antihistamines with seizure events in young children?

**Findings:**

In this cohort study of 3178 children in Korea, prescriptions of a first-generation antihistamine were associated with a 22.0% higher seizure risk in children, especially in those aged 6 to 24 months.

**Meaning:**

These findings reinforce the importance of cautious first-generation antihistamine prescription in young children and underline the need for further research to fully understand associations between antihistamine use and seizure risk.

## Teaser

This cohort study uses a case-crossover design to investigate the association between prescription of first-generation antihistamines and seizures in young children using a nationwide population-based claims dataset.

## Introduction

First-generation H1 antihistamines, developed in the 1940s and 1950s, initially served as tranquilizers and antipsychotics by crossing the blood-brain barrier (BBB) and suppressing histamine neurotransmission in the central nervous system (CNS).^1^ Despite reduced therapeutic use owing to their poor selectivity and interactions with other receptors, these drugs are still widely used for managing rhinorrhea in the common cold^2,3^ or to control an itching sensation^4,5^ in children. Because first-generation antihistamines can cross the BBB, their effects may extend beyond somnolence and drowsiness^1^ to markedly influence brain wave activity.^6^ Thus, caution is advised when prescribing these antihistamines to children younger than 2 years, an age group for whom drug safety data are lacking and first-generation antihistamines are generally not recommended.^7^

Numerous studies^6,8,9,10^ have indicated that first-generation antihistamines can affect brain waves. In particular, they can induce symptomatic seizures, affect electroencephalographic (EEG) activity and seizure thresholds in adults with inherent seizure susceptibility, and alter resting EEG activity.^6,8^ Animal studies have indicated that H1 antihistamines increase seizure susceptibility in rodents,^9^ and studies using genetically modified animal models have suggested a link with epileptic seizures.^10^ Clinically, antihistamines have been identified as a common trigger of acute symptomatic seizures,^11^ and there have been some instances of seizure induction by second-generation antihistamines.^12,13^ Altered seizure patterns were noted in children with febrile seizures who were exposed to antihistamines.^14,15^ However, the impact of first-generation antihistamines on brain waves and the heightened sensitivity to them among vulnerable age groups remains less explored in clinical practice.^11,16^

We hypothesized that acute prescription of first-generation antihistamines increases the risk of seizures, especially in young children. Therefore, this study investigated the association between prescription of first-generation antihistamines and seizures in young children by using a nationwide population-based claims dataset. In this study, children served as their own controls, eliminating time-invariant confounding factors and focusing on the age groups vulnerable to seizures owing to the transient nature of CNS development in children.

## Methods

### Study Design and Data Source

This retrospective cohort study used representative data from the National Health Insurance Service (NHIS) database in Korea, which covers the country’s entire population. Data for individuals born in Korea between January 1, 2002, and December 31, 2005, and followed up until December 31, 2019, or until participants became ineligible for health care insurance were considered. The NHIS database offers essential demographic details, such as birth date, sex, insurance premium, and region of residence. It also includes information on use of health care services, hospital visit types, *International Statistical Classification of Diseases and Related Health Problems*,* Tenth Revision *(*ICD-10*), diagnosis codes, prescribed medication codes, and procedure codes. Only deidentified individual data were used, adhering to the National Health Insurance Act^17^ and ethical guidelines. The study protocol was reviewed and approved by the Institutional Review Board of the Korea National Institute for Bioethics Policy, which waived the need for informed consent because of the use of deidentified data. This study followed the Strengthening the Reporting of Observational Studies in Epidemiology (STROBE) guideline.

### Case-Crossover Design

A case-crossover approach was adopted to assess the risk of seizure events associated with first-generation antihistamine prescription (eFigure in Supplement 1). It analyzes short-term exposure effects on acute events by comparing transient exposure histories within the same individual.^4,17,18^ We chose this design because antihistamines are typically used for short periods, and seizures are considered their immediate health impacts. In the case-crossover design, each participant acts as their own control. This approach inherently eliminates confounding factors that remain constant among individuals. Because the same participants’ data were considered for both the case and control periods, issues related to differential recall were potentially reduced. However, one must be cautious about bias arising from temporal changes in disease severity.

The date of occurrence of a seizure event was marked as the index date. Because the 95th percentile duration of antihistamine prescriptions ranged from 13 to 18 days, the main observational window for first-generation antihistamine prescription was assessed within the 15 days preceding this index date, referred to as the hazard period. This was compared with prescriptions 31 to 45 days and 61 to 75 days before the index date, which served as control periods 1 and 2, respectively. These control periods were presumed to be unrelated to seizures. The findings are reported in accordance with the recommended guidelines for observational studies that use routinely collected health data.^19^

### Study Population

**Figure 1.**
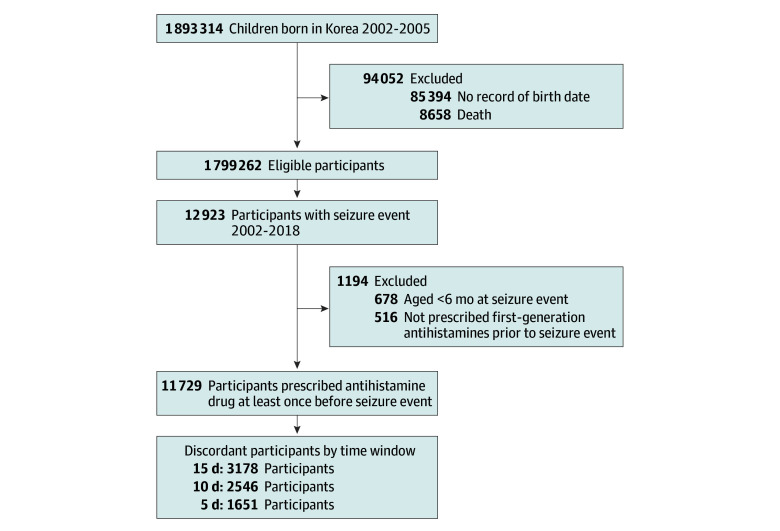
Flowchart of Participant Selection Children were born in Korea from January 1, 2002, to December 31, 2005. A seizure event of interest in this study was defined as an emergency department visit with a principal diagnosis of epilepsy, status epilepticus, or convulsion.

The study population is shown in Figure 1. The data of 1 893 314 children born in Korea between 2002 and 2005 were analyzed. From this cohort, children whose birthdate records were missing and those who died were excluded. The focus of the study was on children who had a seizure event, totaling 12 923 individuals. Additionally, those who had a seizure event at younger than 6 months (n = 678) and those who had not been prescribed first-generation antihistamines before the seizure event (n = 516) were excluded. The final analysis included only those children who were prescribed first-generation antihistamines prior to their seizure event, amounting to 11 729 individuals.

### Seizure Event

The seizure events of interest were defined as emergency department visits with a principal diagnosis of epilepsy (*ICD-10* code G40.X), status epilepticus (*ICD-10* code G41.X), or convulsions (*ICD-10* code R56.8), with each individual’s first event being designed as the index date. Due to the complexities associated with analyzing multiple events from the same individual in a case-crossover study, we focused our analysis on the initial event for each participant. This approach was chosen to prevent overlapping exposure windows and to ensure the independence of observations, essential for the reliability of our statistical analysis.

### Prescription of First-Generation Antihistamines

The focus of this study was on prescription of first-generation antihistamines, that is, pure first-generation antihistamines and compound medications containing first-generation antihistamines. These prescriptions were identified using prescription codes from the NHIS database. The first-generation antihistamines included chlorpheniramine maleate, mequitazine, oxatomide, piprinhydrinate, and hydroxyzine hydrochloride, as listed in eTable 1 in Supplement 1. All drugs were approved by the Korean Food and Drug Administration. Prescription of first-generation antihistamines was defined as having been prescribed these drugs during the case or control period for at least 1 day. Additionally, the participants were classified as new users of first-generation antihistamines if they had not been exposed to these drugs in the 16 to 30 days preceding the index date.

### Covariates

Participants were classified into 3 age groups at the index date: 6 to 24 months, 25 months to 6 years, and 7 years or older. Participants’ birth regions were classified into 4 categories: Seoul, metropolitan areas (Busan, Daegu, Incheon, Gwangju, Daejeon, and Ulsan), urban areas, and rural areas. Economic status was estimated based on insurance payments.

To assess clinical conditions, cases of hospitalization in an intensive care unit before the age of 6 months were identified. Additionally, perinatal conditions were determined using *ICD-10* codes, including length of gestation and fetal growth (*ICD-10* codes P05.X-P08.X), convulsions and CNS disturbances (*ICD-10* codes P90.X-P91.X), birth trauma (*ICD-10* codes P10.X-P15.X), respiratory and cardiovascular disorders specific to the perinatal period (*ICD-10* codes P20.X-P29.X), infections specific to the perinatal period (*ICD-10* codes P35.X-P39.X), congenital malformations (*ICD-10* codes Q00.X-Q89.X), and chromosomal anomalies (*ICD-10* codes Q90.X-Q99.X). If a participant was diagnosed with 1 or more of these conditions, they were considered to have a perinatal condition.

### Statistical Analysis

Data were analyzed from June 3, 2023, to January 30, 2024. In the case-crossover design analysis, the focus was on participants who were exposed to antihistamines during either the hazard period or a control period but not both (ie, discordant participants). These individuals contributed to the odds ratio (OR) estimation. Odds ratios were calculated to assess the association between antihistamine prescription and seizures. This was achieved by comparing the exposure status of the participants during the hazard period with their exposure status during the control periods.

The risks of seizure event with antihistamine prescriptions were analyzed using multivariable conditional logistic regression, with results estimated by ORs and 95% CIs. The analysis was adjusted for various factors, including age, sex, residential area, economic status, season of the index date, and perinatal conditions.^20^ Since the missing data were less than 2% only for region of birth and economic status, the analysis was conducted without imputing the missing data.

For sensitivity analysis, the risk of antihistamine prescription for seizure events was determined using alternative time windows of 10 or 5 days. Hence, the hazard period was defined as 1 to 10 days before the seizure event, with the corresponding control periods set at 31 to 40 or 61 to 70 days before the event, or as 1 to 5 days before the seizure event, with the corresponding control periods set at 31 to 35 or 61 to 65 days before the event. Additionally, to match the seasons of the hazard and control periods, the risk was analyzed by considering 1 to 15 days before the index date as the hazard period and 15 days before the start of a 365-day period before the index date as the control period. In addition, to adjust for clinical conditions that could potentially be associated with the occurrence of seizures, the 9 most common concomitant diseases during the risk and control periods were defined as time-dependent covariates for each participant. These diseases included acute nasopharyngitis, acute pharyngitis, acute tonsillitis, acute sinusitis, acute purulent otitis media, acute upper respiratory tract infection, acute bronchitis, acute bronchiolitis, and asthma. The risk of seizure with antihistamine prescriptions was further analyzed by adjusting for these time-varying covariates.

We also evaluated whether comparable results were obtained when focusing solely on first-generation single-formulation antihistamines and excluding compound medications with first-generation antihistamines. Subgroup analyses were performed by stratifying participants’ characteristics, including age group at the index date, sex, region of birth, economic status, calendar year of birth, season of the index date, and perinatal conditions. For intrasubgroup OR comparisons, the log-OR difference between strata was used to calculate *z* scores and *P* values. Two-sided *P* < .05 indicated statistical significance. This analysis was conducted using the LOGISTIC procedure in SAS software, version 9.4 (SAS Institute Inc).

## Results

### Baseline Characteristics of the Study Population

Baseline demographic and clinical characteristics of the children are presented in Table 1. Among the 11 729 eligible participants who had a seizure event, 3178 (27.1%) were discordant participants who were prescribed antihistamines during either the hazard or the control period. At the index date, 985 participants (31.0%) were aged 6 to 24 months; 1445 (45.5%), 25 months to 6 years; and 748 (23.5%), 7 years or older). The proportion of boys (1776 [55.9%]) was slightly higher than that of girls (1402 [44.1%]). The economic status of the participants was categorized as low (687 [21.6%]), intermediate (1826 [57.5%]), or high (604 [19.0%]). In addition, approximately one-third of the participants (950 [29.9%]) were diagnosed with a perinatal condition. Congenital malformations were the most common perinatal condition, affecting 703 participants (22.1%).

**Table 1.** Baseline Characteristics of the Study Population at the Index Date With 15 Days of Window Period^a^

Variable	Participants, No. % (n = 3178)
Age at the index date^b^	
6-24 mo	985 (31.0)
25 mo to 6 y	1445 (45.5)
≥7 y	748 (23.5)
Sex	
Boys	1776 (55.9)
Girls	1402 (44.1)
Region of birth^c^	
Seoul	1095 (34.5)
Metropolitan	487 (15.3)
Urban	1319 (41.5)
Rural	233 (7.3)
Economic status^d^	
Low (<25th percentile)	687 (21.6)
Intermediate (25th to 75th percentile)	1826 (57.5)
High (>75th percentile)	604 (19.0)
Calendar year at birth	
2002	659 (20.7)
2003	758 (23.9)
2004	863 (27.2)
2005	898 (28.3)
Clinical conditions	
Admission to intensive care unit before age 6 mo	101 (3.2)
Any perinatal condition	950 (29.9)
Length of gestation and fetal growth	84 (2.6)
Convulsion, CNS disturbance	38 (1.2)
Congenital malformation	703 (22.1)
Chromosomal anomaly	50 (1.6)
Birth trauma	14 (0.4)
Respiratory and cardiovascular disorder	97 (3.1)
Infection specific to the perinatal period	242 (7.6)
No. of perinatal conditions	
0	2228 (70.1)
1	714 (22.5)
2	155 (4.9)
≥3	81 (2.5)

Abbreviation: CNS, central nervous system.

^a^ The final study population included participants with a 15-day window who had been prescribed antihistamines during either the hazard or control periods.

^b^ The index date was defined as the date of the seizure event. The seizure event was defined as an emergency department visit with a principal diagnosis of epilepsy (*International Statistical Classification of Diseases and Related Health Problems, Tenth Revision* [*ICD-10*], code G40.X), status epilepticus (*ICD-10* code G41.X), or convulsion (*ICD-10* code R56.8).

^c^ Owing to missing data for 44 participants (1.4%), percentages do not total 100.

^d^ Owing to missing data for 61 participants (1.9%), percentages do not total 100.

### Association Between Antihistamine Prescription and Seizure Events

Table 2 presents the data on antihistamine prescription during different periods before the index date and its association with seizure events. Focusing on the time window of 1 to 15 days, the number of participants who were prescribed antihistamines was 1476 (46.4%) during the hazard period compared with 1239 (39.0%) during control period 1 and 1278 (40.2%) during control period 2. Consequently, the risk of seizure events within 15 days of antihistamine prescription was found to be elevated (adjusted OR [AOR], 1.22 [95% CI, 1.13-1.31]). Notably, new users of antihistamines during the hazard period also demonstrated a higher risk of seizures (AOR, 1.25 [95% CI, 1.14-1.35]) compared with those in the control periods.

**Table 2.** Seizure Event Risk Associated With First-Generation Antihistamine Use

Time window	Total No. of children	Exposed, No. (%)			OR (95% CI)	
		Hazard period	Control period 1	Control period 2	Crude^a^	Adjusted^b^
Before index date						
1-15 d^c^	3178	1476 (46.4)	1239 (39.0)	1278 (40.2)	1.22 (1.14-1.32)	1.22 (1.13-1.31)
Sensitivity analysis						
1-10 d^c^	2546	1150 (45.2)	940 (36.9)	958 (37.6)	1.26 (1.16-1.37)	1.25 (1.15-1.36)
1-5 d^c^	1651	735 (44.5)	563 (34.1)	556 (33.7)	1.36 (1.23-1.50)	1.36 (1.23-1.51)
1-15 d^d^	1597	850 (53.2)	747 (46.8)	NA	1.14 (1.03-1.26)	1.13 (1.02-1.25)

Abbreviations: NA, not applicable; OR, odds ratio.

^a^ Calculated by multivariable conditional logistic regression to assess the association between first-generation antihistamine use and seizure event.

^b^ Adjusted for various factors, including age, sex, residential area, economic status, season at the index date, and perinatal conditions.

^c^ The hazard period was the time window just before the index date, while control periods 1 and 2 were the same duration, starting at 31 and 61 days before the index date, respectively.

^d^ The hazard period was the time window just before the index date, while the control periods were the same duration, starting 1 year before the index date.

In the sensitivity analyses in which the time window was set to either 1 to 10 days or 1 to 5 days, the elevated risk of seizures associated with antihistamine prescription showed similar patterns. Specifically, AORs were 1.25 (95% CI, 1.15-1.36) for the 10-day window and 1.36 (95% CI, 1.23-1.51) for the 5-day window. Moreover, even when further adjusted for concomitant diseases as a time-varying covariate, antihistamine prescriptions significantly increased the risk of seizure events in the 15-day window (AOR, 1.10 [95% CI, 1.01-1.20]), the 10-day window (AOR, 1.13 [95% CI, 1.03-1.25]), and the 5-day window (AOR, 1.21 [95% CI, 1.08-1.36]). In addition, when we focused solely on prescription of first-generation single-formulation antihistamines, excluding compound medications, the results remained consistent with the main findings across the 15-, 10-, and 5-day windows (eTable 2 in Supplement 1).

### Association Between Antihistamine Prescription and Seizure Events According to Participant Characteristics

**Figure 2.**
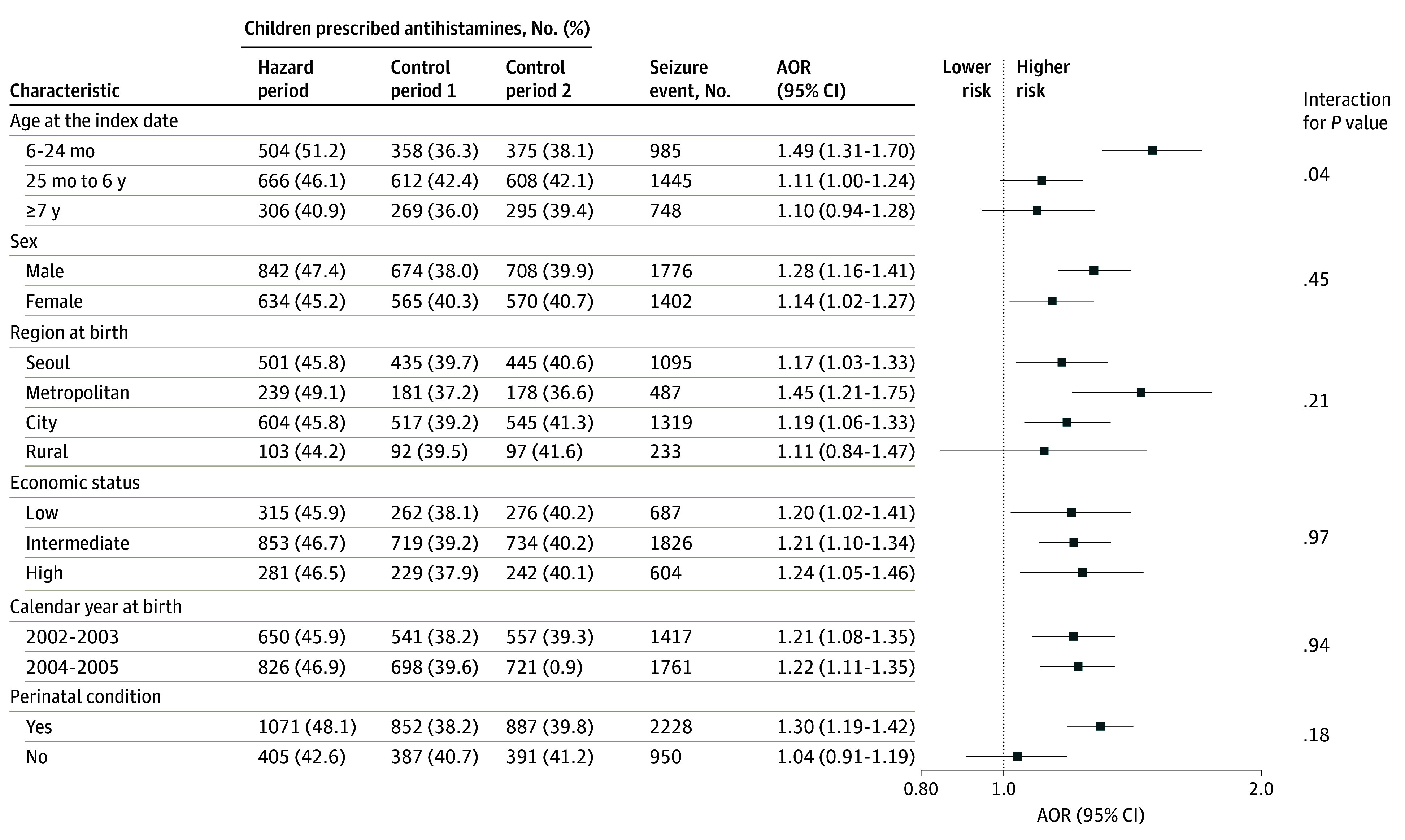
Subgroup Analysis of the Risk for Seizure Events Associated With First-Generation Antihistamines AOR indicates adjusted odds ratio.

Figure 2 illustrates the risk of seizure events associated with antihistamine prescription, broken down by participant characteristics. The analysis revealed interaction effects between subgroups based on age groups at the index date (*P* = .04 for interaction). Notably, children aged 6 to 24 months with an index date had a significantly increased risk, with an AOR of 1.49 (95% CI, 1.31-1.70). However, for children aged 25 months to 6 years who had a seizure event, the AOR was 1.11 (95% CI, 1.00-1.24), and for 7 years or older, the AOR was 1.10 (95% CI, 0.94-1.28).

## Discussion

In this cohort study, we used large-scale national data and a self-controlled case-crossover design to analyze the association between first-generation antihistamine prescription and seizure events. Interestingly, we found that first-generation antihistamine prescription was linked to a 22.0% increase in seizures among children aged 6 to 24 months. This increased risk was consistently observed across stratified analyses and remained consistent when considering different time-control points and medication types. Notably, the study underscores a substantial increase in seizure risk associated with antihistamine prescription among children aged 6 to 24 months. We are not aware of any other studies that have pointed out the increased risk of seizures with first-generation antihistamines in this particular age group, in contrast to older children, for whom we found no such association.

Previous studies^11,12,14,15^ have raised concerns about the potential of antihistamines to induce seizures, especially in acute symptomatic cases and patients with epilepsy and new-onset seizures, and EEG analyses support these findings.^8^ This effect might be mediated by histamine receptors distributed in the brain’s cortex and hippocampus^21,22^ and the ability of antihistamines to cross the BBB, affecting CNS activity.^23,24^ Studies in H1 receptor knockout mice and genetically modified animal models of temporal lobe epilepsy showing increased histidine decarboxylase activity,^10,25^ as well as long-term administration of antihistamines resulting in glutamine synthetase dysfunction,^9,26^ indicated that the role of histamine in modulating brain seizure susceptibility, particularly affecting neuronal excitability in the hippocampus. The possible mechanism by which antihistamines may increase the risk of seizures is complex and involves multiple pathways: inhibition of the role of antihistamines in impeding the antiseizure effect^27^; depletion of hypothalamic neural histamine causing neuronal excitability^16^; impairment of glutamine synthase, a key enzyme in glutamate metabolism and γ-aminobutyric acid synthesis^9^; and direct inhibition of neuronal channels.^28^ Consequently, antihistamines might increase seizure susceptibility by enhancing neural activity and connectivity through the action of histamine receptors and H1 receptor dysfunction, which plays a critical role in regulating seizure intensity and duration.^8^

Interestingly, children exposed to antihistamines between the ages of 6 and 24 months showed an increased risk of seizures compared with other young children. This period is critical for brain development and is characterized by a primitive brain structure and rapid developmental processes, making it a susceptible phase for future brain development, including cognition and fine motor functions.^29,30^ The vulnerability of the infant brain to antihistamines is partly due to the developing BBB, which continues to evolve in this age group.^31^ In infants, incomplete formation of the BBB leads to increased permeability and a higher risk of drug penetration into the brain tissue. Therefore, antihistamines, which are relatively harmless to adults and older children, might markedly affect infants in a negative manner.^17^ Additionally, underdeveloped metabolic pathways in infants influence drug metabolism and excretion.^32^ Moreover, incomplete neural myelination of the brain in young children, which develops rapidly during early childhood, contributes to seizure susceptibility.^33^ These factors contribute to an increased risk of seizures due to antihistamine use during infancy. Therefore, our finding that antihistamines are linked to seizure risk in children 24 months and younger suggests a potential developmental impact. Our results may suggest the need for careful consideration when prescribing antihistamines to infants or patients prone to seizures. In other words, these findings reinforce the importance of prudent and reasonable administration of first-generation antihistamines to vulnerable infants.

The variation in the risk of seizures associated with antihistamines among different age groups of children is likely related to brain development. We found that the older the children, the less pronounced the risk of seizures with the use of antihistamines. This is probably due to improved metabolism and a more mature brain, which are less affected by first-generation antihistamines.^32,34^ However, antihistamines are available over the counter for children older than 24 months but require a prescription for those 24 months and younger. Since our database does not include over-the-counter purchases, we cannot rule out the possibility that this factor influenced the observed interaction effect among age groups, antihistamine prescriptions, and seizures.

### Strengths and Limitations

This study had several strengths. First, it used a longitudinal design with a large, representative sample of children, allowing for robust and generalizable findings. Second, unlike previous studies that focused on ictal effects in adults or febrile children,^11,15^ our study concentrated on children with epilepsy or nonfebrile seizures, excluding those with febrile seizures.

It is essential to recognize the limitations of this study. First, seizure information was obtained from primary diagnoses using insurance data, which did not include details on seizure symptoms. Moreover, we only observed children visiting the emergency department; hence, we could not include those treated in the pediatric outpatient clinic. Second, we could not verify the actual intake of prescribed first-generation antihistamines due to the nature of our database. Third, unmeasured clinical conditions during hazard periods might affect our findings.^35,36,37^ To address these limitations, we sought to confirm our results by adjusting for concomitant infectious diseases as a time-dependent variable.

## Conclusions

In this cohort study, we found an association between antihistamines and an increased odds of seizure events, especially in children aged 6 to 24 months and vulnerable groups. The benefits and risks of antihistamine use should always be carefully considered, especially when prescribing H1 antihistamines to vulnerable infants. Further research is needed to elucidate associations between antihistamine prescriptions and seizure risk.

## Notes

Supplement 1. **eFigure.** Graphic Presentation of the Case-Crossover Design **eTable 1.** Prescription Codes of Antihistamine Drugs **eTable 2.** Seizure Event Risk Associated With First-Generation Antihistamine Use Only

Supplement 2.
Data Sharing Statement
