# Supplementary material for: First-Generation Antihistamines and Seizures in Young Children
Source: JAMA Netw Open. 2024 Aug 28;7(8):e2429654. doi: 10.1001/jamanetworkopen.2024.29654 (PMC11358850; doi:10.1001/jamanetworkopen.2024.29654)
Supplement: Supplement 1. — eFigure. Graphic Presentation of the Case-Crossover Design eTable 1. Prescription Codes of Antihistamine Drugs eTable 2. Seizure Event Risk Associated With First-Generation Antihistamine Use Only [file jamanetwopen-e2429654-s001.pdf]

Supplementary Online Content

Kim JH, Ha EK, Han B, et al. First-generation antihistamines and seizures in young children. *JAMA Netw Open*. 2024;7(8):e2429654. doi:10.1001/jamanetworkopen.2024.29654

eFigure. Graphic Presentation of the Case-Crossover Design

eTable 1. Prescription Codes of Antihistamine Drugs

eTable 2. Seizure Event Risk Associated With First-Generation Antihistamine Use Only

This supplementary material has been provided by the authors to give readers additional information about their work.

© 2024 Kim JH et al. *JAMA Network Open*.

eFigure. Graphic Presentation of the Case-Crossover Design

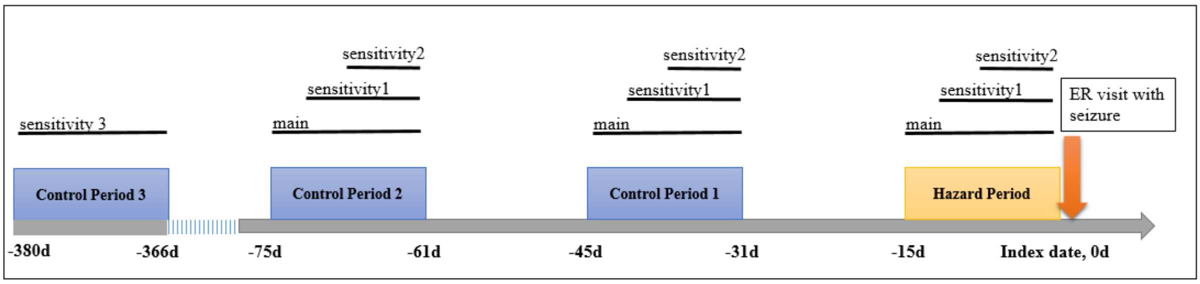

The observation period was comprised of a hazard period and two control periods. The figure primarily demonstrates that the time windows were 15 days, in comparison to 30 and 60 days prior to the index date. Arrows signify the date of first-generation antihistamine exposure, the index date, and day 0. Sensitivity analysis was conducted using window periods of 10 and 5 days. The final sensitivity analysis involved a comparison with the period 1-year prior.

© 2024 Kim JH et al. *JAMA Network Open*.

eTable 1. Prescription Codes of Antihistamine Drugs

	Prescription code	General name	Prescribed event
**First generation antihistamines**			101,929,428
	1318	Chlorpheniramine maleate	49,231,510
	1905	Mequitazine	25,422,440
	2062	Oxatomide	251,842
	2133	Piprinhydrinate	17,327,415
	1721	Hydroxyzine hydrochloride	8,741,404
	2119	Phenylephrine hydrochloride	0
	1193	Buclizine 2HCl	275
	1393	Cyproheptadine	219,822
	1459	Dimenhydrinate	528,937
	1692	Homochlorcyclizine	180,268
	2116	Pheniramine maleate	10
	2191	Promethazine	25,505
**Compound medication including first generation antihistamines**			202,840
	5305	Chlorpheniramine maleate + Phenylephrine hydrochloride	5,974
	5306		1,940
	5307		1,901
	5308		102,748
	5309		55,943
	5302		0
	5303		0
	5310	Pseudoephedrine hydrochloride + Triprolidine hydrochloride	1,202
	5311		134
	5312		39
	5313		5,883
	5314		372
	5315		25,963
	5316		741
	5324	Dextromethorphan+Pseudoephedrine+ Triprolidine hydrochloride	0

© 2024 Kim JH et al. *JAMA Network Open*.

eTable 2. Seizure Event Risk Associated With First-Generation Antihistamine Use Only

Time window before the index date	Total N	N (%)			Crude OR (95% CI)[b]	Adjusted OR (95% CI)[c]
		Hazard period	Control period 1	Control period 2		
1–15 days[a]	3,164	1,470	1,233	1,273	1.361 (1.232-1.504)	1.364 (1.232-1.509)
1–10 days[a]	2,534	1,145	934	954	1.263 (1.164-1.369)	1.253 (1.153-1.360)
1–5 days[a]	1,644	732	562	553	1.361 (1.232-1.504)	1.364 (1.232-1.509)

Abbreviations: CI, confidence interval; N, number; OR, odds ratio.
[a] The hazard period is the time window just before the index date, while the control periods are the same duration, starting 31 and 61 days before the index date.
[b] Crude ORs calculated by multivariable conditional logistic regression to assess the association between first-generation antihistamine and seizure events.
[c] Adjusted OR was adjusted for various factors including age, sex, residential area, economic status, season at the index date, and perinatal conditions.

© 2024 Kim JH et al. *JAMA Network Open*.
