# Supplementary material for: First-Generation Antihistamines and Seizures in Young Children
Source: JAMA Netw Open. 2024 Aug 28;7(8):e2429654. doi: 10.1001/jamanetworkopen.2024.29654 (PMC11358850; doi:10.1001/jamanetworkopen.2024.29654)
Supplement: Supplement 2. — Data Sharing Statement [file jamanetwopen-e2429654-s002.pdf]

Data Sharing Statement

Kim. First-Generation Antihistamines and Seizures in Young Children. *JAMA Netw Open*. Published August 28, 2024. doi:10.1001/jamanetworkopen.2024.29654

Data
Data available: No

Additional Information
Explanation for why data not available: This study was based on the National Health Claims Database established by the National Health Insurance Service of the Republic of Korea. Applications for using the National Health Insurance Service data are reviewed by the Inquiry Committee of Research Support; if the application is approved, raw data are provided to the applicant for a fee. We cannot provide access to the data, analytic methods, and research materials to other researchers because of the intellectual property rights of this database owned by the National Health Insurance Corporation. However, investigators who wish to reproduce our results or replicate the procedure can use the database, which is open for research purposes (https://nhiss.nhis.or.kr/ accessed on 2023).
